# Air Pressure, Humidity and Stroke Occurrence: A Systematic Review and Meta-Analysis

**DOI:** 10.3390/ijerph13070675

**Published:** 2016-07-05

**Authors:** Yongjun Cao, Xia Wang, Danni Zheng, Thompson Robinson, Daqing Hong, Sarah Richtering, Tzen Hugh Leong, Abdul Salam, Craig Anderson, Maree L. Hackett

**Affiliations:** 1 Department of Neurology, The Second Affiliated Hospital of Soochow University, No.1055, Sanxiang Rd., Suzhou 215004, China; yongjuncao@126.com; 2 The George Institute for Global Health, P.O. Box M201, Missenden Road, Sydney, NSW 2050, Australia; dzheng@georgeinstitute.org.au (D.Z.); srichtering@georgeinstitute.org.au (S.R.); hugh-96@hotmail.com (T.H.L.); asalam@georgeinstitute.org.au (A.S.); canderson@georgeinstitute.org.au (C.A.); mhackett@georgeinstitute.org.au (M.L.H.); 3 Sydney Medical School, The University of Sydney, Sydney, NSW 2050, Australia; 4 Department of Cardiovascular Sciences and NIHR Biomedical Research Unit for Cardiovascular Diseases, University of Leicester, Leicester LE1 7RH, UK; tgr2@leicester.ac.uk; 5 Division of Nephrology, Sichuan Academy of Medical Sciences & Sichuan Provincial People’s Hospital, Chengdu 610072, China; hongdaqing11@126.com; 6 Department of Neurology, Royal Prince Alfred Hospital, Syndey, NSW 2050, Australia; 7 College of Health and Wellbeing, The University of Central Lancashire, Preston PR1 2HE, UK

**Keywords:** stroke, weather, air pressure, humidity, systematic review

## Abstract

Background/Aims: An influence of climate upon stroke risk is biologically plausible and supported by epidemiological evidence. We aimed to determine whether air pressure (AP) and humidity are associated with hospital stroke admission. Methods: We searched MEDLINE, Embase, PsycINFO, CINAHL, Web of Science, and GEOBASE, from inception to 16 October 2015 to identify relevant population-based observational studies. Where possible, data were pooled for meta-analysis with odds ratios (OR) and corresponding 95% confidence intervals (CI) by means of the random-effect method. Results: We included 11 studies with a total of 314,385 patients. The effect of AP was varied across studies for ischemic stroke (IS) and subarachnoid haemorrhage (SAH). Pooled ORs (95%CI) associated with 1 hPa increase in AP for the risk of IS, intracerebral hemorrhage (ICH) and SAH were 1.00 (0.99–1.01), 1.01 (0.99–1.02) and 1.02 (0.97–1.07) respectively. The pooled ORs (95%CI) associated with 1 percent increase in humidity for the risk of IS and ICH were 1.00 (1.00–1.01) and 1.00 (0.99–1.01) respectively. Conclusion: This review shows that there is no evidence of a relationship between AP or humidity and the occurrence of hospital admission for stroke. Further research is needed to clarify the extent and nature of any relationship between AP, humidity and stroke in different geographical areas.

## 1. Introduction

Estimates from the Global Burden of Diseases, Injuries, and Risk Factors Study (GBD 2010) ranked stroke as the second most common cause of death [1] and the third most common cause of disability-adjusted life-years [2] worldwide in 2010. To date, over 300 traditional cardiovascular risk factors have been reported; the five most important being tobacco use, alcohol use, hypertension, high cholesterol levels and obesity [3]. While these traditional risk factors are fundamental in explaining the etiology and prognosis of cerebrovascular diseases, their relative importance may alter with time [4]. It is possible that changes in external stimuli, such as ambient temperature, atmospheric pressure (AP) and humidity, are relevant, and may help explain why stroke is more likely to occur at particular times, and in particular geographical regions [5,6].

An influence of climate upon stroke risk is biologically plausible and supported by epidemiological evidence [3,7]. Investigations into these relationships are important as they could yield public health strategies to help protect the vulnerable from adverse environmental conditions, for example, cold and heat waves [3]. Though there have been extensive studies investigating the association between ambient temperature and stroke risk [3,8,9], the significance of other meteorological risk factors, such as AP and humidity, has been less frequently explored. The results from several studies on AP and humidity have been inconsistent: a 1965 Japanese study involving 74 subjects [10], reported that intracerebral hemorrhage tended to occur when humidity was low (<40%), and AP was raised. In contrast, a hospital-based study in Mexico showed no apparent relationship between AP and stroke occurrence [11].

We conducted a systematic review of population-based stroke studies to summarize and appraise evidence on the relationship between AP and humidity and hospital stroke admission.

## 2. Methods

The protocol for this study was registered with the international prospective register of systematic reviews (PROSPERO)—CRD42015026364. The systematic review was reported following Meta-analysis of Observational Studies in Epidemiology (MOOSE) guidelines [12].

### 2.1. Study Eligibility Criteria

Population or community-based, and hospital registry studies that explicitly stated consecutive patient recruitment from within clearly defined geographical boundaries and of a minimum one-year duration (to ensure all seasons were represented) were included. Patients aged 18 years and over, of any race or gender with a clinical or imaging (computed tomography (CT) or magnetic resonance imaging (MRI)) diagnosis of first-ever or recurrent stroke, regardless of pathological subtype, were included. We excluded hospital-based studies and studies with broad vascular outcomes, no specific results for stroke, with less than 50% stroke patients, or with fewer than 100 patients. In addition, studies that cited weather data from unofficial sources (e.g., any weather website) were excluded. There were no language restrictions set.

### 2.2. Databases and Sources

A comprehensive search strategy (Supplementary Material 1), developed in consultation with a university librarian, neurologists, and epidemiologists, was used to address the unique features and indexing of each of the five electronic databases (MEDLINE, Embase, PsycINFO, CINAHL, and Web of Science), that were searched from inception to 16 October 2015. GEOBASE was also searched to capture any relevant studies that might have been published in the geographical/meteorological rather than the medical literature.

In order to capture important ‘‘grey literature’’, the websites of the following organizations were searched for relevant reports: World Health Organization; European Union; Health Effects Institute (USA); Environmental Protection Agency (USA); National Institutes of Health (USA); Department of Health (UK); Department for Environment, Food, and Rural Affairs (UK); Department of Health (Australia); Department of the Environment (Australia); Ministry of Health of the People’s Republic of China; Ministry of Environmental Protection of the People’s Republic of China. The reference lists of any relevant reviews appearing in their reports were examined to search for original studies.

### 2.3. Data Collection and Extraction

Xia Wang scrutinized the titles and abstracts, and excluded clearly irrelevant references; this process was checked by Sarah Richtering. Xia Wang and Daqing Hong extracted data independently from the included studies using EpiData; any disagreements were resolved by a third reviewer (Danni Zheng).

### 2.4. Data Analysis

Six items were used to define quality criteria to appraise included studies [13]: (A) presence of clear hypotheses; (B) prospective study design; (C) description of the population, at least including its size, and the gender ratio; (D) stroke assessed by CT, MRI or angiography, cerebrospinal fluid examination or autopsy; (E) a clear description of the meteorological determinants investigated, when possible including the unit of measurement; and (F) description of other risk factors for stroke. Articles were defined as ‘high quality’ when at least five of these criteria were satisfied.

We pooled the results of studies in which an effect estimate was presented as a regression coefficient, percentage change (PC), relative risk (RR) or odds ratio (OR). We converted the regression coefficient and PC to RR using the equations: RR = $e^{\beta }$ and RR = 1 + PC, respectively. Thereafter, all RRs were converted to ORs using the equation: OR = RR/[(1 − P0) + (P0 × RR)], where P0 = the incidence in the non-exposed group. Because population stroke incidence fulfilled a Poisson distribution, this indicated a small probability event and we assumed OR = RR. The degree of heterogeneity was calculated using the *I*^2^-index, and *I*^2^ > 50% indicated heterogeneity. ORs from the individual studies were pooled using the random effects method because of the large degree of variation in the effect estimates between studies. Otherwise, a narrative review of studies was presented. Estimates of association were reported separately for each stroke type.

## 3. Results

**Figure 1:**
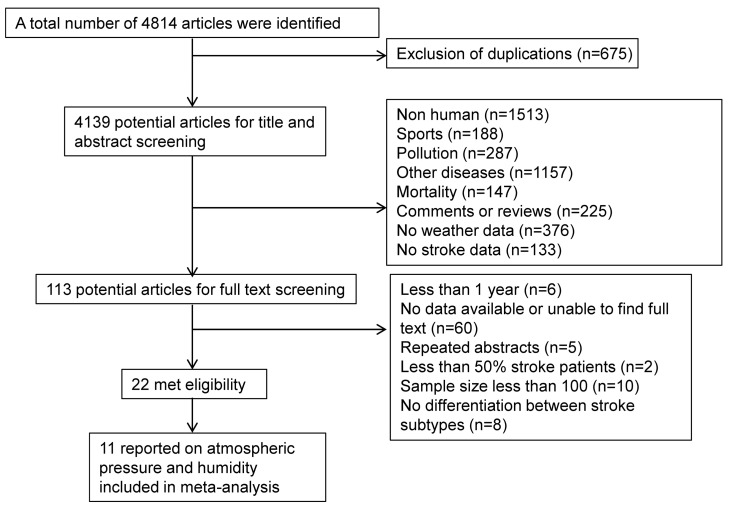
Flow chart of literature search.

Of 4814 references obtained after execution of the search strategy, 113 were found to be relevant after screening titles and abstracts (Figure 1). Twenty-one studies satisfied the eligibility criteria, and eleven that reported AP/humidity data (314,385 stroke patients) were included in the review. A summary of the included studies’ characteristics is presented in Table 1. In brief, studies were conducted in ten countries, and across five continents in the northern hemisphere. Three studies were from countries with latitude under 30 degrees, and ten were between 30 to 60 degrees. Of the 13 studies, two were community-based, 8 were population-based, and three were based on stroke registries.

Four studies [9,14,15,16] were defined as high quality as they met five of the quality criteria, another three [8,17,18] met four of the quality criteria and the remainder met less than four of the quality criteria. Only three studies [9,17,19] were prospectively designed. And only four studies [9,14,15,16] described stroke risk factors distribution in the reported population.

### 3.1. Ischemic Stroke

#### 3.1.1. Air Pressure

**Figure 2:**
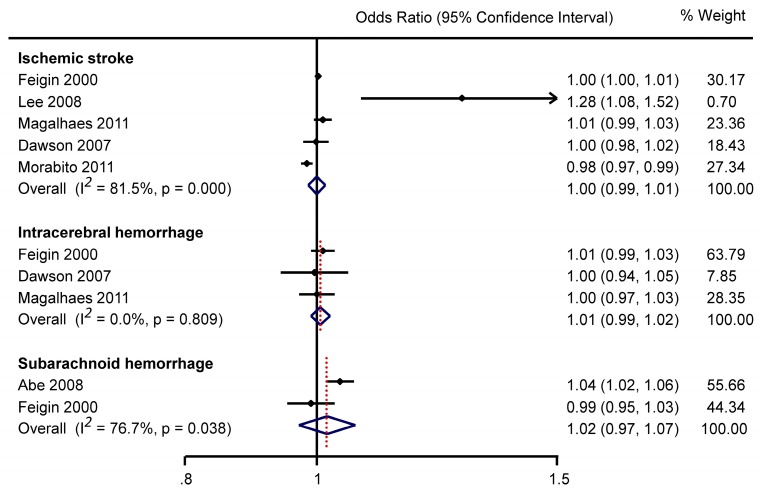
Meta analysis of mean daily air pressure and stroke with odds ratios qantifying the association between every one hPa increase in air pressure and stroke occurrence.

Six studies reporting data on mean AP, with 222,764 IS patients, are summarized in online Table S1. Pooled estimate (of five studies) showed no significant association between AP and IS (OR 1.00, 95%CI 0.99–1.01) (Figure 2).

Two of three studies reported a non-significant association between AP changes and ischemic stroke (IS) risk (online Table S2). Jimenez-Conde et al. [17] observed a significant relationship between the incidence of non-lacunar stroke and AP changes compared with the previous day.

#### 3.1.2. Humidity

**Figure 3:**
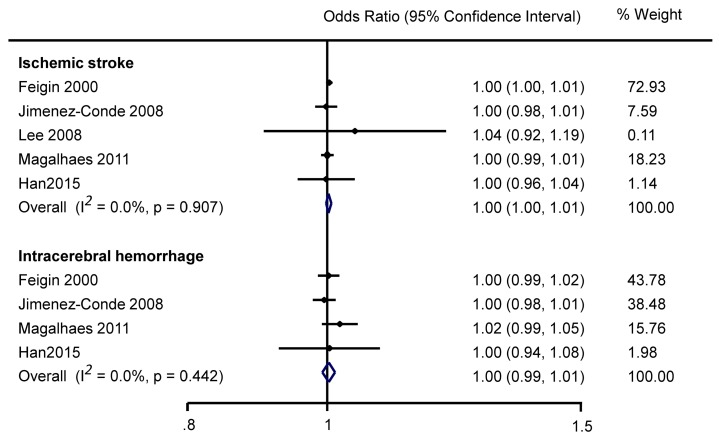
Meta analysis of mean daily humidity and stroke with odds ratios quantifying the association between every one percent increase in humidity and stroke occurrence.

Five studies (online Table S3) showed no significant association between humidity and IS (pooled estimate OR 1.00, 95%CI 1.00–1.01; Figure 3).

### 3.2. Intracerebral Hemorrhage

#### 3.2.1. Air Pressure

Three studies (online Table S4) involving a total of 972 ICH patients assessed the association between AP and ICH risk. None of the individual studies reported a significant association, and the pooled estimate was OR 1.01, 95%CI 0.99–1.02 (Figure 2).

However, a statistically significant increase in ICH risk with greater AP change (in the previous 24 or 48 h) was reported in two of the three studies (online Table S5).

#### 3.2.2. Humidity

No study demonstrated a significant association between humidity and ICH, whether analysing mean humidity as a continuous variable (online Table S6), categorical variable (online Table S7) or using pooled estimate (Figure 3).

### 3.3. Subarachnoid Hemorrhage

#### 3.3.1. Air Pressure

In a Japanese population study, Abe et al. [14] reported that high mean AP on the onset day was associated with a higher subarachnoid haemorrhage (SAH) risk (online Table S8), whilst Feigin et al. [9] reported a non-significant association. Overall, the pooled estimate of two studies was OR 1.02, 95%CI 0.97–1.07 (Figure 2). There was similar inconsistency in the two studies investigating an association between AP change over the previous 24 h and SAH risk (online Table S9); one study reporting a larger AP reduction was associated with SAH risk [19], the other did not find a significant relationship [9].

#### 3.3.2. Humidity

One [20] of two studies [20,22] reported a significant association between mean monthly humidity and SAH occurrence. Of studies [9,19] reporting the relationship between humidity change over the previous 24 h and SAH risk, one [19] reported that a larger humidity reduction was associated with SAH risk (online Table S10).

## 4. Discussion

This systematic review shows no association between daily mean AP and humidity with IS, ICH and SAH occurrence. This review included only population-based studies so that the association could be assessed in a representative population.

It is of great importance to conduct this review in a population-based study with at least 1 year recruitment period, as current evidence from studies investigating the relationship between AP [8,9,16], humidity [9,16,23] and stroke are inconsistent in different types of study design. A clinical stroke registry in Glasgow, UK of 5723 IS and 666 ICH patients from 1990 to 2005 reported a relationship between falling AP and ICH [8]. A Japanese study with only 6-month recruitment period reported that the combination of air temperature, variation of temperature gradient, variation of relative humidity and AP gradient influenced IS and SAH mortality rates [24,25]. Whilst consistent with two other hospital-based studies suggesting that AP and humidity fluctuations are associated with increased stroke rates [11,23], two well conducted population-based studies in Russia [9] and Portugal [16] failed to show such a relationship.

It has been proposed that changes in AP may exert stress on atherosclerotic plaques, resulting in plaque rupture [26]. A heat stroke rat model found tissue damage during exercise in hot and humid environments, that was related to inflammation, oxidative stress and apoptosis [27]. High humidity may also cause dehydration, which can increase thrombotic risk [28]. However, those influence may be not as strong as some other weather conditions such as temperature which may be responsible for physiological changes that could increase stroke risk. These include increases in blood pressure, erythrocyte and thrombocyte counts, and blood viscosity seen in cold weather [29]. Plasma fibrinogen concentration has also been shown to be higher in older patients, especially in cold weather [30]. This may partly explain why AP and humidity are not associated with stroke occurrence.

Several limitations to this review should be acknowledged. Firstly, we were not able to assess publication bias since a limited number of studies were included. Secondly, many included studies had small sample sizes, and results were not adjusted for confounders. Some of the pooled results show significant heterogeneity. Thirdly, although we assessed the quality of the included articles, we were unable to incorporate quality scores in our meta-analysis because of the overall poor reporting of results. Finally, it was not possible to compare data between different populations because of a lack of standardized methods and diagnostic criteria [7]. This reinforces the need for locale-specific data to better understand the effects of weather on stroke occurrence.

## 5. Conclusions

In conclusion, this review shows that there is no evidence of a relationship between AP or humidity on the occurrence of hospital admission for stroke. Further research is needed to clarify the extent and nature of any relationship between AP, humidity and stroke in different geographical areas.

## Figures and Tables

**Table 1:** Characteristics of studies included in the review.

Author and Year of Publication	Title	Location	Latitude	Year(s) of Study	Sample Size	Age (mean, y) Female (%)	Only First-Ever Stroke	Stroke Subtype	Study Type	Study Quality
Abe 2008 [14]	Effects of meteorological factors on the onset of subarachnoid hemorrhage: a time-series analysis	Japan, Tokyo	35.6833° N	2005	1729	63.3 Female (60%)	No	SAH	Population study	ACDEF
Dawson 2008 [8]	Associations between meteorological variables and acute stroke hospital admissions in the west of Scotland	United Kingdom, Glasgow	55.8580° N	1990–2005	6389	71.2 Female (53%)	No	IS and ICH	Stroke registry	ACDE
Feigin 2000 [9]	A population-based study of the associations of stroke occurrence with weather parameters in Siberia, Russia (1982–1992)	Russia, Siberia	61.0137° N	1982–1992	2208	Age range: 25–74 Female (57%)	Yes	IS, ICH and SAH	Stroke registry	ABCEF
Han 2015 [15]	Effect of seasonal and monthly variation in weather and air pollution factors on stroke incidence in Seoul, Korea	South Korea, Seoul	37.5667° N	2004–2013	3001	Age >19 Female (49%)	No	IS and ICH	Stroke registry	ACDEF
Jimenez-Conde 2008 [17]	Weather as a trigger of stroke: daily meteorological factors and incidence of stroke subtypes	Spain, Barcelona	41.3833° N	2001–2003	1286	Not reported	No	IS and ICH	Population	ABDE
Lai 2014 [20]	The association between meteorological parameters and aneurysmal subarachnoid hemorrhage: a nationwide analysis	USA, 41 states	38.8833° N	2001–2010	16,970	Median: 53 (IQR 34-72)	No	SAH	Population	ADE
Lee 2008 [21]	Seasonal variation in ischemic stroke incidence and association with climate, a six-year population-based study	Taiwan	23.6978° N	1998–2003	168,977	Age range: 20–84	No	IS	Population	AE
Lejeune 1994 [19]	Association of occurrence of aneurysmal bleeding with meteorological variations in the north of france	France, North France region	47.0000° N	1989–1991	283	49.1 Female (53%)	No	SAH	Community	ABE
Magalhaes 2011 [16]	Are stroke occurrence and outcome related to weather parameters? Results from a population-based study in Northern Portugal	Portugal, Porto	41.1621° N	1998–2000	462	All ages Female (62%)	Yes	IS and ICH	Stroke registry	ACDEF
Morabito 2011 [18]	Innovative approaches helpful to enhance knowledge on weather-related stroke events over a wide geographical area and a large population	Italy, Tuscany	43.3500° N	1997–2007	112,870	All ages	No	IS, ICH and SAH	Hospital registry	ACDE
Oyoshi 1999 [22]	Relationship between aneurysmal subarachnoid hemorrhage and climatic conditions in the subtropical region, Amami-Oshima, in Japan	Japan, Amami-Oshima	28.2500° N	1986–1996	210	All ages, 64.3	No	SAH	Hospital registry	AE

Abbreviations: IS, ischemic stroke; ICH, intracerebral hemorrhage; SAH, subarachnoid hemorrhage; N, north; IQR, interquartile range; (A) presence of clear hypotheses; (B) prospective study design; (C) description of the population, at least including its size, and the gender ratio; (D) stroke assessed by CT, MRI or angiography, cerebrospinal fluid examination or autopsy; (E) a clear description of the meteorological determinants investigated, when possible including the unit of measurement; and (F) description of other risk factors for stroke.

## Supplementary Materials

The following are available online at www.mdpi.com/1660-4601/13/7/675/s1, Supplementary Material 1. Search strategy. Table S1: Summary characteristics of studies included in meta-analysis for mean air pressure and ischemic stroke, Table S2: Summary characteristics of studies reporting air pressure change and ischemic stroke, Table S3: Summary characteristics of studies included in meta-analysis for relative humidity and ischemic stroke, Table S4: Summary characteristics of studies included in meta analysis for mean air pressure and intracerebral hemorrhage, Table S5: Summary characteristics of studies reporting air pressure change and intracerebral hemorrhage, Table S6: Summary characteristics of studies included in meta anlysis for mean humidity and intracerebral hemorrhage, Table S7: Summary characteristics of studies included in systematic review for mean humidity and intracerebral hemorrhage, Table S8: Summary characteristics of studies included in meta analysis for mean air pressure and subarachnoid hemorrhage, Table S9: Summary characteristics of studies included in systematic review for air pressure change and subarachnoid hemorrhage, Table S10: Summary characteristics of studies included in systematic review for humidity and subarachnoid hemorrhage.

## References

[1] Lozano R., Naghavi M., Foreman K., Lim S., Shibuya K., Aboyans V., Abraham J., Adair T., Aggarwal R., Ahn S.Y. (2012). Global and regional mortality from 235 causes of death for 20 age groups in 1990 and 2010: A systematic analysis for the Global Burden of Disease study 2010. Lancet.

[2] Murray C.J., Vos T., Lozano R., Naghavi M., Flaxman A.D., Michaud C., Ezzati M., Shibuya K., Salomon J.A., Abdalla S. (2012). Disability-adjusted life years (DALYs) for 291 diseases and injuries in 21 regions, 1990–2010: A systematic analysis for the global burden of disease study 2010. Lancet.

[3] McArthur K., Dawson J., Walters M. (2010). What is it with the weather and stroke?. Expert Rev. Neurother..

[4] Berginer V.M., Goldsmith J., Batz U., Vardi H., Shapiro Y. (1989). Clustering of strokes in association with meteorologic factors in the Negev desert of Israel: 1981–1983. Stroke.

[5] Feigin V.L., Anderson C.S., Rodgers A., Bennett D.A. (2002). Subarachnoid haemorrhage occurrence exhibits a temporal pattern—Evidence from meta-analysis. Eur. J. Neurol..

[6] Smolensky M.H., Portaluppi F., Manfredini R., Hermida R.C., Tiseo R., Sackett-Lundeen L.L., Haus E.L. (2015). Diurnal and twenty-four hour patterning of human diseases: Cardiac, vascular, and respiratory diseases, conditions, and syndromes. Sleep Med. Rev..

[7] Feigin V.L., Wiebers D.O. (1997). Environmental factors and stroke: A selective review. J. Stroke Cerebrovasc. Dis..

[8] Dawson J., Weir C., Wright F., Bryden C., Aslanyan S., Lees K., Bird W., Walters M. (2008). Associations between meteorological variables and acute stroke hospital admissions in the west of scotland. Acta Neurol. Scand..

[9] Feigin V.L., Nikitin Y.P., Bots M.L., Vinogradova T.E., Grobbee D.E. (2000). A population-based study of the associations of stroke occurrence with weather parameters in siberia, russia (1982–1992). Eur. J. Neurol..

[10] Ono Y., Horibe H., Hayakawa N., Aoki N., Okada H. (1970). Biometeorologie studies on cerebrovascular diseases. (IV). Evaluation of meterorologic factors, their changes or combinations on the occurrence of cerebrovascular accident. Jpn. Circ. J..

[11] Olivares L., Castaneda E., Grife A., Alter M. (1973). Risk factors in stroke: A clinical study in mexican patients. Stroke.

[12] Stroup D.F., Berlin J.A., Morton S.C., Olkin I., Williamson G.D., Rennie D., Moher D., Becker B.J., Sipe T.A., Thacker S.B. (2000). Meta-analysis of observational studies in epidemiology: A proposal for reporting. Meta-analysis of observational studies in epidemiology (MOOSE) group. JAMA.

[13] De Steenhuijsen Piters W.A., Algra A., van den Broek M.F., Dorhout Mees S.M., Rinkel G.J. (2013). Seasonal and meteorological determinants of aneurysmal subarachnoid hemorrhage: A systematic review and meta-analysis. J. Neurol..

[14] Abe T., Ohde S., Ishimatsu S., Ogata H., Hasegawa T., Nakamura T., Tokuda Y. (2008). Effects of meteorological factors on the onset of subarachnoid hemorrhage: A time-series analysis. J. Clin. Neurosci..

[15] Han M.H., Yi H.J., Kim Y.S., Kim Y.S. (2015). Effect of seasonal and monthly variation in weather and air pollution factors on stroke incidence in Seoul, Korea. Stroke.

[16] Magalhaes R., Silva M.C., Correia M., Bailey T. (2011). Are stroke occurrence and outcome related to weather parameters? Results from a population-based study in northern Portugal. Cerebrovasc. Dis..

[17] Jimenez-Conde J., Ois A., Gomis M., Rodriguez-Campello A., Cuadrado-Godia E., Subirana I., Roquer J. (2008). Weather as a trigger of stroke. Daily meteorological factors and incidence of stroke subtypes. Cerebrovasc. Dis..

[18] Morabito M., Crisci A., Vallorani R., Modesti P.A., Gensini G.F., Orlandini S. (2011). Innovative approaches helpful to enhance knowledge on weather-related stroke events over a wide geographical area and a large population. Stroke.

[19] Lejeune J.P., Vinchon M., Amouyel P., Escartin T., Escartin D., Christiaens J.L. (1994). Association of occurrence of aneurysmal bleeding with meteorologic variations in the north of France. Stroke.

[20] Lai P.M., Dasenbrock H., Du R. (2014). The association between meteorological parameters and aneurysmal subarachnoid hemorrhage: A nationwide analysis. PLoS ONE.

[21] Lee H.C., Hu C.J., Chen C.S., Lin H.C. (2008). Seasonal variation in ischemic stroke incidence and association with climate: A six-year population-based study. Chronobiol. Int..

[22] Oyoshi T., Nakayama M., Kuratsu J. (1999). Relationship between aneurysmal subarachnoid hemorrhage and climatic conditions in the subtropical region, Amami-oshima, in Japan. Neurol. Medico-Chir..

[23] Bokonjic R., Zec N. (1968). Strokes and the weather. A quantitative statistical study. J. Neurol. Sci..

[24] Ono Y. (1969). Biometeorologic studies on cerebrovascular diseases. I. Effects of meteorologic factors on the death from cerebrovascular accident. Jpn. Circ. J..

[25] Ono Y., Aoki N., Horibe H., Hayakawa N., Okada H. (1974). Biometeorologic studies on cerebrovascular diseases. V. A multivariate analysis of meteorologic effects on cerebrovascular accident. Jpn. Circ. J..

[26] Houck P.D., Lethen J.E., Riggs M.W., Gantt D.S., Dehmer G.J. (2005). Relation of atmospheric pressure changes and the occurrences of acute myocardial infarction and stroke. Am. J. Cardiol..

[27] Li D., Wang X., Liu B., Liu Y., Zeng Z., Lu L., Zheng Z., Li B., Zheng Z. (2014). Exercises in hot and humid environment caused liver injury in a rat model. PLoS ONE.

[28] Lockett L.J. (2012). Hydration-dehydration, heat, humidity, and “cool, clear, water”. Sports Med. Arthrosc. Rev..

[29] Keatinge W.R., Coleshaw S.R., Cotter F., Mattock M., Murphy M., Chelliah R. (1984). Increases in platelet and red cell counts, blood viscosity, and arterial pressure during mild surface cooling: Factors in mortality from coronary and cerebral thrombosis in winter. Br. Med. J..

[30] Woodhouse P.R., Khaw K.T., Plummer M., Foley A., Meade T.W. (1994). Seasonal variations of plasma fibrinogen and factor vii activity in the elderly: Winter infections and death from cardiovascular disease. Lancet.

