# Supplementary material for: Air Pressure, Humidity and Stroke Occurrence: A Systematic Review and Meta-Analysis
Source: Int J Environ Res Public Health. 2016 Jul 5;13(7):675. doi: 10.3390/ijerph13070675 (PMC4962216; doi:10.3390/ijerph13070675)
Supplement: Supplementary file 1 [file ijerph-13-00675-s001.pdf]

Supplementary Materials: Air Pressure, Humidity and Stroke Occurrence: A Systematic Review and Meta-Analysis

Yongjun Cao, Xia Wang, Danni Zheng, Thompson Robinson, Daqing Hong, Sarah Richtering, Tzen Hugh Leong, Abdul Salam, Craig Anderson and Maree L Hackett

Supplementary Material 1 Search Strategy

MEDLINE

Stroke

1. cerebrovascular disorders/or exp basal ganglia cerebrovascular disease/or exp brain ischemia/or exp carotid artery diseases/or exp intracranial arterial diseases/or exp intracranial arteriovenous malformations/or exp "intracranial embolism and thrombosis"/or exp intracranial hemorrhages/or stroke/or exp brain infarction/or vasospasm, intracranial/or vertebral artery dissection/
2. (stroke$ or poststroke or post-stroke or cerebrovasc$ or brain vasc$ or cerebral vasc$ or cva$ or apoplex$ or SAH).tw.
3. ((brain$ or cerebr$ or cerebell$ or intracran$ or intracerebral) adj3 (isch?emi$ or infarct$ or thrombo$ or emboli$ or occlus$)).tw.
4. ((brain$ or cerebr$ or cerebell$ or intracerebral or intracranial or subarachnoid) adj3 (h?emorrhag$ or h?ematoma$ or bleed$)).tw.
5. exp paresis/ or exp Gait Disorders, Neurologic/
6. (hemipleg$ or hemipar$ or pares?s or paretic).tw.
7. 1 or 2 or 3 or 4 or 5 or 6

Weather

8. exp weather/or exp climate/or exp meteorological concepts/
9. weather or climate
10. (climat$ or season$ or humid$ or latitud$).tw.
11. (temperature$ adj3 (hot$ or cold$ or ambient or extreme$ or atmospher$ or variation$ or chang$ or absolute or range$ or fall$ or rise$ or fluctuat$)).tw
12. ((atmospher$ or air or barometric) adj3 pressur$).tw
13. (snow$ or sun$ or rain$ or freez$ or precipitation$ or meteorolog$).tw.
14. 8 or 9 or 10 or 11 or 12 or 13

Study type

15. Epidemiologic studies/or Exp case control studies/or Exp cohort studies/or Case control.tw. or (cohort adj (study or studies)).tw. or Cohort analy$.tw. or (Follow up adj (study or studies)).tw. or (observational adj (study or studies)).tw. or Longitudinal.tw. or Retrospective.tw. or Cross sectional.tw. or Cross-sectional studies/
16. Human/
17. 7 and14 and 15 and 16

EMBASE

Stroke

1. 'cerebrovascular disease' or 'basal ganglion hemorrhage'/exp or 'brain hematoma'/exp or 'brain hemorrhage'/exp or 'brain infarction'/exp or 'brain ischemia'/exp or 'carotid artery disease'/exp or 'cerebral artery disease' or 'cerebrovascular accident'/exp or 'intracranial aneurysm'/exp or 'occlusive cerebrovascular disease'/exp or 'stroke unit' or 'stroke patient'
2. (stroke$ or poststroke or post-stroke or cerebrovasc$ or brain vasc$ or cerebral vasc$ or cva$ or apoplex$ or SAH):ab or (stroke$ or poststroke or post-stroke or cerebrovasc$ or brain vasc$ or cerebral vasc$ or cva$ or apoplex$ or SAH):ti
3. ((brain$ or cerebr$ or cerebell$ or intracran$ or intracerebral) near/3 (isch?emi$ or infarct$ or thrombo$ or emboli$ or occlus$)):ti or ((brain$ or cerebr$ or cerebell$ or intracran$ or intracerebral) near/3 (isch?emi$ or infarct$ or thrombo$ or emboli$ or occlus$)):ab
4. ((brain$ or cerebr$ or cerebell$ or intracerebral or intracranial or subarachnoid) near/3 (h?emorrhag$ or h?ematoma$ or bleed$)):ti or ((brain$ or cerebr$ or cerebell$ or intracerebral or intracranial or subarachnoid) near/5 (h?emorrhag$ or h?ematoma$ or bleed$)):ab
5. 'paresis'/exp or 'neurologic gait disorder'/exp
6. (hemipleg$ or hemipar$ or paresis or pareses or paretic):ab or (hemipleg$ or hemipar$ or paresis or pareses or paretic):ti
7. 1 or 2 or 3 or 4 or 5 or 6

Weather

8. 'weather'/exp or 'climate'/exp or 'season'/exp or 'air temperature'/exp or 'environmental temperature'/exp or 'high temperature'/exp or 'low temperature'/exp or 'atmospheric pressure'/exp or 'humidity'/exp or weather or climate or meteorological phenomena
9. (climat$ or season$ or humid$ or latitud$):ab or (climat$ or season$ or humid$ or latitud$):ti
10. (temperature$ near/3 (hot$ or cold$ or ambient or extreme$ or atmospher$ or variation$ or chang$ or absolute or range$ or fall$ or rise$ or fluctuat$)):ab or (temperature$ near/3 (hot$ or cold$ or ambient or extreme$ or atmospher$ or variation$ or chang$ or absolute or range$ or fall$ or rise$ or fluctuat$)):ti
11. ((atmospher$ or air or barometric) near/3 pressure):ab or ((atmospher$ or air or barometric) near/3 pressure):ti
12. (snow$ or sun$ or rain$ or freez$ or precipitation$ or meteorolog$):ab or (snow$ or sun$ or rain$ or freez$ or precipitation$ or meteorolog$):ti
13. 8 or 9 or 10 or 11 or 12

Study type

14. 'clinical study' or 'case control study' or 'family study' or 'longitudinal study' or 'retrospective study'
15. 'prospective study' not 'randomized controlled trials'
16. 'cohort'
17. Cohort near (analysis or study or studies)
18. (Case control near (study or studies)):ab or (Case control near (study or studies)):ti
19. (follow up near (study or studies)):ab or (follow up near (study or studies)):ti
20. (observational near (study or studies)):ab or (observational near (study or studies)):ti
21. (epidemiologic$ near (study or studies)):ab or (epidemiologic$ near (study or studies)):ti
22. (cross sectional near (study or studies)):ab or (cross sectional near (study or studies)):ti
23. 14 or 15 or 16 or or 17 or 18 or 19 0r 20 or 21 or 22

CINAHL

Stroke

1. Cerebrovascular Disorders OR (MH "Basal Ganglia Cerebrovascular Disease+") OR (MH "Carotid Artery Diseases+") OR (MH "Cerebral Ischemia+") OR (MH "Arteriovenous Malformations+") OR (MH "Cerebral Vasospasm") OR (MH "Intracranial Arterial Diseases+") OR (MH "Intracranial Embolism and Thrombosis+") OR (MH "Intracranial Hemorrhage+") OR (MH "Stroke+") OR (MH "Vertebral Artery Dissections") or (MH "Stroke Patients") OR (MH "Stroke Units")
2. TI (stroke* or poststroke or post-stroke or cerebrovasc* or brain vasc* or cerebral vasc* or cva* or apople* or SAH) or AB (stroke* or poststroke or post-stroke or cerebrovasc* or brain vasc* or cerebral vasc* or cva* or apople* or SAH)
3. (TI (brain or cerebr* or cerebell* or intracran* or intracerebral) or AB (brain or cerebr* or cerebell* or intracran* or intracerebral)) and (TI (ischemi* or ischaemi* or infarct* or thrombo* or emboli* or occlus*) or AB (ischemi* or ischaemi* or infarct* or thrombo* or emboli* or occlus*))
4. (TI (brain* or cerebr* or cerebell* or intracerebral or intracranial or subarachnoid) or AB (brain* or cerebr* or cerebell* or intracerebral or intracranial or subarachnoid)) and (TI (haemorrhage* or hemorrhage* or haematoma* or hematoma* or bleed*) or AB (haemorrhage* or hemorrhage* or haematoma* or hematoma* or bleed*))
5. TI (hemipleg* or hemipar* or paresis or pareses or paretic) or AB (hemipleg* or hemipar* or paresis or pareses or paretic)
6. 1 or 2 or 3 or 4 or 5 or 6

Weather

7. (MH "Meteorological Factors+") or (MH "atmosphere+") OR (MH "Atmospheric Pressure+") or (MH "Weather+") OR (MH "Temperature+"))
8. (MM "Cold") OR (MM "Heat") OR (MM "Humidity") OR (MM "Rain") OR (MM "Snow") or (MM "Seasons")
9. AB (climat$ or season$ or humid$ or latitud$) or TI (climat$ or season$ or humid$ or latitud$)
10. AB (temperature$ N3 (hot$ or cold$ or ambient or extreme$ or atmospher$ or variation$ or chang$ or absolute or range$ or fall$ or rise$ or fluctuat$)) or TI (temperature$ N3 (hot$ or cold$ or ambient or extreme$ or atmospher$ or variation$ or chang$ or absolute or range$ or fall$ or rise$ or fluctuat$))
11. TI ((atmospher$ or air or barometric) N3 pressure) or AB ((atmospher$ or air or barometric) N3 pressure)
12. TI (snow$ or sun$ or rain$ or freez$ or precipitation or meteorolog$) or AB (snow$ or sun$ or rain$ or freez$ or precipitation$ or meteorolog$)
13. 7 OR 8 OR 9 OR 10 OR 11 OR 12

Study type

14. Prospective studies OR (MH "Case Control Studies+") or Correlational studies or Nonconcurrent prospective studies or Cross sectional studies or TI (cohort N (study or studies)) OR AB (cohort N (study or studies)) OR TI (observational N (study or studies)) OR AB (observational N (study or studies))

Web of science

Stroke

1. TS=(stroke OR cva OR cerebrovascular OR "cerebral vascular" OR hemipleg* OR paresis OR pareses OR hemipares* OR parapares*)
2. TS=(paretic OR hemiparetic OR paraparetic OR dystoni*)
3. TS=((cerebral OR cerebellar OR brain* OR vertebrobasilar) NEAR/3 (infarct* OR isch$emi* OR thrombo* OR emboli* OR apoplexy))
4. TS=((cerebral OR brain* OR subarachnoid) NEAR/3 (haemorrhage OR haemorrhage OR haematoma OR hematoma OR bleed*))
5. #4 OR #3 OR #2 OR #1

Weather

6. TS=(weather or climat* or season* or humid* or latitud* or snow* or rain* or precipitation*)
7. TS=(temperature* NEAR/3 (hot* or cold* or ambient or extreme* or atmospher* or variation* or chang* or absolute or range* or fall$ or rise$ or fluctuat$))
8. TS=((atmospher* or air or barometric) NEAR/3 pressur*)
9. #6 OR #7 OR #8
10. #5 and #9

PsycINFO (Ovid)

Stroke

1. cerebrovascular disorders/ or cerebral hemorrhage/ or exp cerebral ischemia/ or cerebral small vessel disease/ or cerebrovascular accidents/ or subarachnoid hemorrhage/
2. (stroke or poststroke or post-stroke or cerebrovasc$ or brain vasc$ or cerebral vasc$ or cva$ or apoplex$ or SAH).tw.
3. ((brain$ or cerebr$ or cerebell$ or intracran$ or intracerebral) adj3 (isch?emi$ or infarct$ or thrombo$ or emboli$ or occlus$)).tw.
4. ((brain$ or cerebr$ or cerebell$ or intracerebral or intracranial or subarachnoid) adj3 (h?emorrhage$ or h?ematoma$ or bleed$)).tw.
5. hemiparesis/ or hemiplegia/
6. (hemipleg$ or hemipar$ or paresis or paretic).tw.
7. or/1-6

Weather

8. exp seasonal variations/ exp Atmospheric Conditions/
9. (weather or climat$ or season$ or humid$ or latitud$).tw.
10. (temperature$ adj3 (hot$ or cold$ or ambient or extreme$ or atmospher$ or variation$ or chang$ or absolute or range$ or fall$ or rise$ or fluctuat$)).tw
11. ((atmospher$ or air or barometric) adj3 pressur$).tw
12. (snow$ or sun$ or rain$ or freez$ or precipitation$ or meteorolog$).tw.
13. 8 or 9 or 10 or 11 or 12 or 13
14. 7 and 14

Geobase

Stroke

1. (({nervous system disorder} WN CV OR {nervous system disorder} WN RGI) OR ({stroke} WN CV OR {stroke} WN RGI))
2. ((stroke$ or poststroke or post-stroke or cerebrovasc$ or brain vasc$ or cerebral vasc$ or cva$ or apoplex$ or SAH) WN KY)
3. $brain near/3 $ischemi or $brain near/3 $ischaemi or $brain near/3 $infarct or $brain near/3 $thrombo or $brain near/3 $emboli or $brain near/3 $occlus
4. $cerebr near/3 $ischemi or $cerebr near/3 $ischaemi or $cerebr near/3 $infarct or $cerebr near/3 $thrombo or $cerebr near/3 $emboli or $cerebr near/3 $occlus

5. $cerebell near/3 $ischemi or $cerebell near/3 $ischaemi or $cerebell near/3 $infarct or $cerebell near/3 $thrombo or $cerebell near/3 $emboli or $cerebell near/3 $occlus
6. $intracran near/3 $ischemi or $intracran near/3 $ischaemi or $intracran near/3 $infarct or $intracran near/3 $thrombo or $intracran near/3 $emboli or $intracran near/3 $occlus
7. intracerebral near/3 $ischemi or intracerebral near/3 $ischaemi or intracerebral near/3 $infarct or intracerebral near/3 $thrombo or intracerebral near/3 $emboli or intracerebral near/3 $occlus
8. $brain near/3 $hemorrhage or $cerebr near/3 $hemorrhage or $cerebell near/3 $hemorrhage or $intracran near/3 $hemorrhage or intracerebral near/3 $hemorrhage
9. $brain near/3 $ hematoma or $cerebr near/3 $ hematoma or $cerebell near/3 $ hematoma or $intracran near/3 $ hematoma or intracerebral near/3 $hematoma
10. $brain near/3 $bleed or $cerebr near/3 $bleed or $cerebell near/3 $bleed or $intracran near/3 $bleed or intracerebral near/3 $bleed
11. ((hemipleg$ or hemipar$ or pares?s or paretic or Neurologic Gait Disorders) WN KY)

Weather

12. (({atmospheric and meteorological phenomena} WN CV OR {atmospheric and meteorological phenomena} WN RGI) OR ({climate} WN CV OR {climate} WN RGI) OR ({weather} WN CV OR {weather} WN RGI) OR ({severe weather} WN CV OR {severe weather} WN RGI))
13. ((weather or climat$ or season$ or humid$ or latitud$) WN KY)
14. (({air temperature} WN CV OR {air temperature} WN RGI))
15. (({atmospheric pressure} WN CV OR {atmospheric pressure} WN RGI))
16. (({season} WN CV OR {season} WN RGI) OR ({seasonality} WN CV OR {seasonality} WN RGI))
17. ((snow or sun or rain or freeze or precipitation) WN KY)

Table S1. Summary characteristics of studies included in meta analysis for mean air pressure and ischaemic stroke.

Author and Year of Publication	Number of IS Cases Reported	Statistical Analysis Method	Adjusting Variables	Reported Estimates	Overall Conclusion
Dawson 2007	5723	Negative binomial model	Time trend, season and day of the week	PC in admissions (95%CI): −1.5% (−3.6%–0.7%)	No statistically significant association
Feigin 2000	1929	Poisson regression	Age, season, geomagnetic and solar activity	RR (95%CI): 0.99 (0.97–1.00)	Statistically significant association
Magalhaes 2011	348	Poisson regression	Not reported	RR (95%CI): 1.01 (0.99–1.02)	No statistically significant association
Morabito 2011	45,787	Poisson generalized linear model	Years, seasons, days of the week, celebrations, summer decrement of population	Estimate (±SE): −0.017 (±0.005)	Statistically significant negative association
Lee 2008	168,977	Auto-regressive integrated moving average	Time trend, season and month	Estimate (±SE): 0.245 (±0.087)	Statistically significant positive association

Abbreviations: IS, ischaemic stroke; PC: percentage change; CI: confidence interval; RR: relative risk; SE: standard error.

Table S2. Summary characteristics of studies reporting air pressure change and ischaemic stroke.

Author and Year of Publication	Number of IS Cases Reported	Variables Studied	Statistical Analysis Method	Adjusting Variables	Reported Estimates	Overall Conclusion
Dawson 2008	5723	Change in AP over the preceding 24 h and 48 h	Negative binomial model	Time trend, season and day of the week	PC in admissions (95%CI): −1.7% (−5.0%–1.7%)	No statistically significant association
Feigin 2000	1929	AP change over the preceding 24 h	Poisson regression	None (univariate model)	Any change towards increasing vs. no change, RR (95%CI): 1.01 (0.89–1.14) Any change towards decreasing vs. no change, RR (95%CI): 1.10 (0.98–1.24)	No statistically significant association
Jimenez-Conde 2008	1043	AP changes over the preceding 24 h	log-linear Poisson	Not explicitly reported	AP falls: RR, 0.940 AP rises: RR, 1.005 AP changes: RR, 1.033	Larger AP falls and smaller AP changes are associated with lower risk of non-lacunar stroke

Abbreviations: AP, atmospheric pressure; IS, ischaemic stroke; PC: percentage change; CI: confidence interval; RR: relative risk

Table S3. Summary characteristics of studies included in meta analysis for relative humidity and ischaemic stroke.

Author and Year of Publication	Number of IS Cases Reported	Statistical Analysis Method	Adjusting Variables	Reported Estimates	Overall Conclusion
Feigin 2000	1929	Poisson regression	None (univariate model)	RR (95%CI): 1.00 (1.00–1.01)	No statistically significant association
Jimenez-Conde 2008	1043	Logistic regression	Not explicitly reported	OR (95%CI): 1.00 (0.98–1.01)	No statistically significant association
Lee 2008	168,977	Auto-regressive integrated moving average	Time trend and season	Estimate (±SE): 0.044 (±0.065)	No statistically significant association
Magalhaes 2011	348	Poisson regression	Not reported	RR (95%CI): 1.00 (0.99–1.01)	No statistically significant association
Han 2015	2202	Logistic regression	Other meteorologic and air pollution parameters, past medical history, age and sex	RR (95%CI): 0.99 (0.95–1.03)	No statistically significant association

Abbreviations: IS, ischaemic stroke; RR: relative risk; OR: odds ratio; SE: standard error; CI: confidence interval.

Table S4. Summary characteristics of studies included in meta analysis for mean air pressure and intracerebral haemorrhage.

Author and Year of Publication	Number of ICH Cases Reported	Statistical Analysis Method	Adjusting Variables	Reported Estimates	Overall Conclusion
Feigin 2000	215	Poisson regression	None (univariate model)	RR (95%CI): 1.01 (0.99–1.03)	No statistically significant association
Dawson 2008	666	Poisson regression	Time trend, season, and day of the week	PC in admissions (95%CI): −4.0% (−9.5%–1.9%) (per 10 hPa)	No statistically significant association
Magalhaes 2011	91	Poisson regression	Not reported	RR (95%CI): 1.00 (0.97–1.03)	No statistically significant association

Abbreviations: ICH, intracerebral haemorrhage; PC: percentage change; CI: confidence interval; RR: relative risk.

Table S5. Summary characteristics of studies reporting air pressure change and intracerebral haemorrhage.

Author and Year of Publication	Number of ICH Cases Reported	Weather Parameter Studied	Analysis Model	Adjusting Variables	Reported Estimates	Overall Conclusion
Dawson 2008	666	AP change over the preceding 24 h and 48 h	Poisson regression	Time trend, season, and day of the week	PC in admissions (95%CI): −1.1% (−9.8%–8.6%) (per 10 hPa change over preceding 24 h) PC in admissions (95%CI): −6.6% (−12.7%–−0.2%) (per 10 hPa change over preceding 48 h)	Significant association between fall in AP over the preceding 48 h with increased rate of ICH
Feigin 2000	215	AP change over the preceding 24 h	Poisson regression	None (univariate model)	Any change towards increasing vs. no change, RR (95%CI): 0.85 (0.58–1.24) Any change towards decreasing vs. no change, RR (95%CI): 1.36 (0.96–1.92)	No statistically significant association
Jimenez-Conde 2008	243	AP changes with respect to the previous day	Logistic regression, log-linear Poisson regression	Not explicitly reported	AP variations: RR: 1.04, *p* value 0.124 AP falls: RR: 0.97, *p* value 0.431 AP rises: RR: 1.07, *p* value 0.027	Significant association between rises in AP and risk of ICH

Abbreviations: AP, atmospheric pressure; IS, ischaemic stroke; ICH, intracerebral haemorrhage; hpa, hectopascals; PC: percentage change; CI: confidence interval; RR: relative risk.

Table S6. Summary characteristics of studies included in meta anlysis for mean humidity and intracerebral haemorrhage.

Author and Year of Publication	Number of ICH Cases Reported	Analysis Model	Adjusting Variables	Reported Estimates	Overall Conclusion
Feigin 2000	215	Poisson regression	None (univariate model)	RR (95%CI): 1.00 (0.98–1.01)	No statistically significant association
Jimenez-Conde 2008	243	Logistic regression	Not explicitly reported	OR (95%CI): 1.00 (0.98–1.01)	No statistically significant association
Magalhaes 2011	91	Poisson regression	Not reported	RR (95%CI): 1.02 (0.99–1.04)	No statistically significant association
Han 2015	799	Poisson generalized linear regression	Temperature range, humidity, PM_{10} and NO_2	RR (95%CI): 1.02 (0.95–1.09) (per 5% increments)	No statistically significant association

Abbreviations: ICH, intracerebral haemorrhage; PC: percentage change; CI: confidence interval; RR: relative risk; OR: odds ratio.

Table S7. Summary characteristics of studies included in systematic review for mean humidity and intracerebral haemorrhage.

Author and Year of Publication	Number of ICH Cases Reported	Weather Parameter Studied	Analysis Model	Adjusting Variables	Reported Estimates	Overall Conclusion
Feigin 2000	215	Mean humidity, categorical: mild (73% to 83%) *vs.* high (>84%), low (<72%) *vs.* high (>84%).	Poisson regression	None (univariate model)	Mild vs high: RR (95%CI): 1.00 (0.98–1.01) Low *vs.* high: RR (95%CI): 1.20 (0.38–3.76)	No statistically significant association

Abbreviations: ICH, intracerebral haemorrhage; CI: confidence interval; RR: relative risk.

Table S8. Summary characteristics of studies included in meta analysis for mean air pressure and subarachnoid haemorrhage.

Author and Year of Publication	Number of SAH Cases Reported	Statistical Analysis Method	Adjusting Variables	Reported Estimates	Overall Conclusion
Abe 2008	1729	Autoregressive integrated moving average model	Temperature and humidity	Estimate (±SE): 0.039 (±0.012)	Statistically significant association between high AP and SAH risk
Feigin 2000	64	Poisson regression	Age, season, geomagnetic and solar activity	RR (95%CI): 0.99 (0.95–1.03)	No statistically significant association

Abbreviation: SAH, subarachnoid aneurysmal haemorrhage; AP, atmospheric pressure; SE: standard error.

Table S9. Summary characteristics of studies included in systematic review for air pressure change and subarachnoid haemorrhage.

Author and Year of Publication	Number of SAH Cases Reported	Weather Variable Studied	Statistical Analysis Method	Adjusting Variables	Reported Estimates	Overall Conclusion
Feigin 2000	64	AP change over the preceding 24 h	Poisson regression	None (univariate model)	Any change towards increasing vs. no change, RR (95%CI): 0.62 (0.32–1.22) Any change towards decreasing vs. no change, RR (95%CI): 0.57 (0.29–1.14)	No statistically significant association
Lejeune, 1994	238	Reduction of 100 Pa of AP on the day before SAH	Logistic regression	Temperature, humidity and sunshine duration	OR (95%CI) 1.02 (1.01–1.02)	AP reduction the day before SAH is associated with higher risk of SAH

Abbreviations: AP, atmospheric pressure; SAH, subarachnoid aneurysmal haemorrhage.

Table S10. Summary characteristics of studies included in systematic review for humidity and subarachnoid haemorrhage.

Author and Year of Publication	Number of SAH Cases Reported	Temperature Variable Studied	Statistical Analysis Method	Adjusting Variables	Reported Estimates	Overall Conclusion
Lai 2014	16,970	Monthly mean humidity	Linear regression	Sex	Estimate (95%CI): −0.041 (−0.05–−0.03)	Statistically significant association between lower relative humidity and increase in SAH admissions
Oyoshi 1999	210	Monthly mean humidity	Pearson correlation	N/A	Correlation coefficient 0.14	No statistically significant association
Feigin 2000	64	Humidity change over the preceding 24 h	Poisson regression	None (univariate model)	Any change towards increasing vs. no change, RR (95%CI): 0.74 (0.39–1.43) Any change towards decreasing vs. no change, RR (95%CI): 0.74 (0.37–1.47)	No statistically significant association
Lejeune, 1994	238	Reduction of 1% of minimal humidity on the day of SAH	Logistic regression	Temperature, air pressure and sunshine duration	OR (95%CI): 1.03 (1.01–1.04)	Reduction in humidity on day of SAH is associated with higher risk of SAH

Abbreviations: SAH, subarachnoid aneurysmal haemorrhage; CI, confidence interval; RR, relative risk.

© 2016 by the authors. Submitted for possible open access publication under the terms and conditions of the Creative Commons Attribution (CC-BY) license (http://creativecommons.org/licenses/by/4.0/).
